# Molecular Dynamics Simulation of Tryptophan Hydroxylase-1: Binding Modes and Free Energy Analysis to Phenylalanine Derivative Inhibitors

**DOI:** 10.3390/ijms14059947

**Published:** 2013-05-10

**Authors:** Hao Zhong, Wei Huang, Gu He, Cheng Peng, Fengbo Wu, Liang Ouyang

**Affiliations:** 1 State Key Laboratory of Biotherapy, West China Hospital, Sichuan University, Chengdu 610041, China; E-Mails: zhonghao88830@163.com (H.Z.); wufengbo_@163.com (F.W.); 2 State Key Laboratory Breeding Base of Systematic Research, Development and Utilization of Chinese Medicine, Chengdu University of Traditional Chinese Medicine, Chengdu 610041, China; E-Mails: huangwei@cdutcm.edu.cn (W.H.); pengchengchengdu@126.com (C.P.)

**Keywords:** tryptophan hydroxylase, phenylalanine derivative, molecular dynamics simulation, MM/GBSA

## Abstract

Serotonin is a neurotransmitter that modulates many central and peripheral functions. Tryptophan hydroxylase-1 (TPH1) is a key enzyme of serotonin synthesis. In the current study, the interaction mechanism of phenylalanine derivative TPH1 inhibitors was investigated using molecular dynamics (MD) simulations, free energy calculations, free energy decomposition analysis and computational alanine scanning. The predicted binding free energies of these complexes are consistent with the experimental data. The analysis of the individual energy terms indicates that although the van der Waals and electrostatics interaction contributions are important in distinguishing the binding affinities of these inhibitors, the electrostatic contribution plays a more crucial role in that. Moreover, it is observed that different configurations of the naphthalene substituent could form different binding patterns with protein, yet lead to similar inhibitory potency. The combination of different molecular modeling techniques is an efficient way to interpret the interaction mechanism of inhibitors and our work could provide valuable information for the TPH1 inhibitor design in the future.

## 1. Introduction

5-hydroxytryptamine (5-HT, serotonin) is a monoamine neurotransmitter. Biochemically derived from tryptophan, serotonin is primarily found in the gastrointestinal (GI) tract, platelets, and in the central nervous system (CNS) of animals including humans [1,2]. In mammals, 5-HT is synthesized from the amino acid l-tryptophan by a short metabolic pathway consisting of two enzymes: tryptophan hydroxylase (TPH), and amino acid decarboxylase (DDC). The TPH-mediated reaction is the rate-limiting step in the pathway [3]. TPH has been found in two forms: TPH1, in several tissues, and TPH2, which is a neuron-specific isoform [4].

In the GI system, TPH1 is primarily expressed and dysregulation of the peripheral 5-HT signaling system is involved in the etiology of several conditions such as functional GI disorders, chemotherapy-induced emesis, and heart valve damage [5,6]. Therefore, it is believed that inhibitors of TPH1 have proven effective in treating a wide range of diseases and disorders associated with the serotonergic systems, such as irritable bowel syndrome [7,8].

Recently, new research has shown that gut-derived 5-HT is a powerful inhibitor of osteoblast proliferation and bone formation [9–11]. Yadav and co-workers reported that a small molecule inhibitor of TPH1 has the potential to become a new class of bone anabolic drugs that can be added to the armamentarium to treat osteoporosis [12,13]. Thus, TPH1 can be considered as a new drug target and this mechanism is totally different from any known anti-osteoporosis drugs [14,15]. In very recent work, 3D-QSAR focusing on phenylalanine series compounds such as TPH1 inhibitors have been reported [16]. In the QSAR study, a combination of the ligand-based and structure-based methods is used to clarify the essential quantitative structure–activity relationship of the known TPH1 inhibitors. To elucidate the protein–ligand interaction at the atomic level of these compounds helps significantly to obtain the TPH1 inhibitors with higher activity. The detailed modes of mechanism of the phenylalanine derivative inhibitor–TPH1 interactions, however, are not entirely understood.

In the present study, computational studies including molecular dynamics (MD) simulations, molecular mechanics generalized Born/surface area (MM/GBSA) binding free energy calculations and decomposition of free energy on a per-residue basis are conducted to deeply explore the molecular basis for the binding. In addition, the computational alanine scanning and the structural analysis are carried out to gain insight into the binding mechanism.

## 2. Results and Discussion

### 2.1. The Dynamics Stability MD Simulation

**Figure 1:**
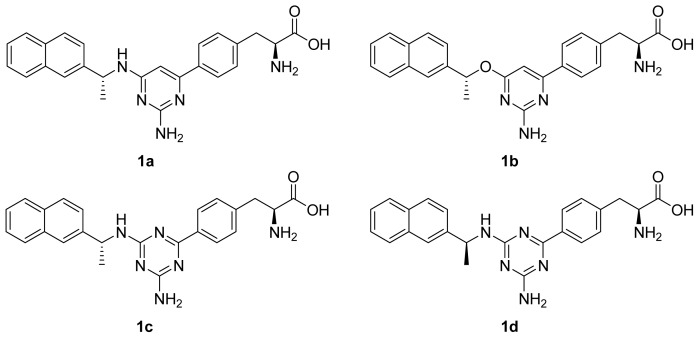
The chemical structures of the phenylalanine derivative inhibitors of TPH1.

**Figure 2:**
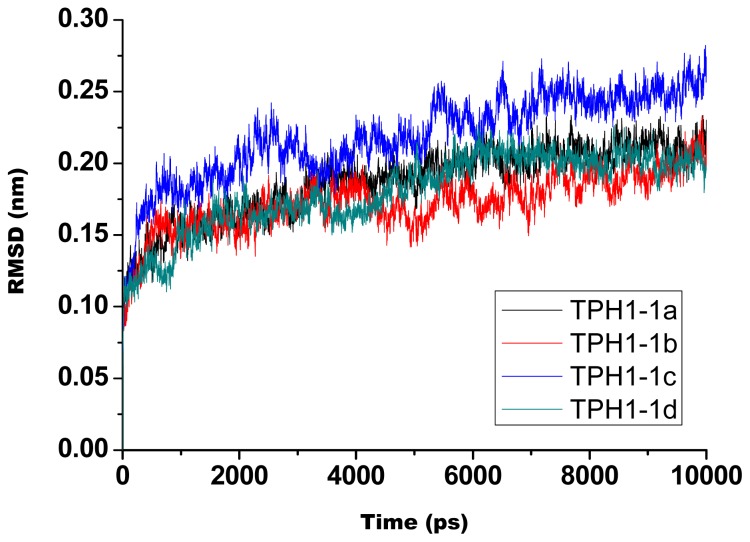
The root-mean-square deviations (RMSD) of the backbone atoms relative to their initial minimized complex structures as a function of time for 1a (black), 1b (red), 1c (blue) and 1d (cyan).

In this study, the MD simulations of four TPH1–inhibitor complexes (showed in Figure 1) were successfully run for 10 ns scale. To evaluate the reliable stability of the MD trajectories and the difference of the stabilities in the MD simulations, there were calculated the RMSD values of the TPH1 backbone atoms relative to the initial minimized structure through the phase of the simulation (plotted in Figure 2). One can see that the 1a- and 1d-TPH1 complexes reached equilibrium after 5 ns of the simulation phase, while the 1b- and 1c-TPH1 complexes were not stable until about 7 ns. According to Figure 2, the RMSD values of the 1a-, 1b-, 1c- and 1d-TPH1 complexes were 0.17, 0.16, 0.19 and 0.23 nm, respectively, with a deviation lower than 0.05 nm; among these structures, the 1a-TPH1 complex had the most reliable stability. These results showed that the trajectories of the MD simulations for the four complexes were stable after 7 ns, so it was reasonable to do the binding free energy calculation and free energy decomposition based on the snapshots extracted from 7 to 10 ns.

**Figure 3:**
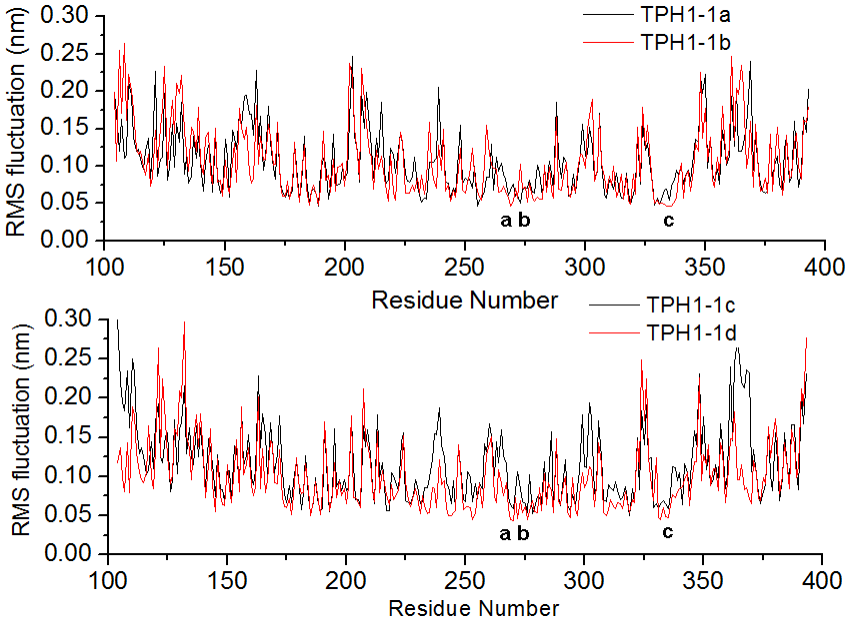
Root-mean-square fluctuation (RMSF) of the backbone atoms (CA, N, C) *versus* residue numbers for the TPH1–inhibitor complexes. The residues a, b and c were Asp269, His272 and Ser336, respectively.

More detailed analysis of root-mean-square fluctuation (RMSF) *versus* the protein residue number for the four complexes is illustrated in Figure 3. In this figure, it is observed that the four inhibitor/protein complexes possess the similar RMSF distributions, indicating that these inhibitors could have the similar interaction mode with TPH1 on the whole. Moreover, the active site regions (such as Asp269, His272, Ser336, *etc.*) show a rigid behavior for all complexes.

To estimate the difference between the MD average structures and crystal structures, the average structures of the MD-simulated complexes from the last 3 ns of MD simulations were superimposed with the crystal structure of TPH-1c complexes (plotted in Figure S1). According to the Figure S1, the MD average structures of four complexes are overall very similar to their crystal structures. However, local conformational differences were also observed. In the case of the TPH-1b and TPH-1d complexes, loop 1 obviously departs from its crystal structure. In the case of the TPH-1a and TPH-1b complexes, loop 2 deviates significantly from its crystal structures. According to Figure S1, the loop 1 and 2 located at the binding site, the binding of inhibitor may lead to slight shifts of the two loops. These results basically agree with the previous RMSD and RMSF analyses.

### 2.2. Calculation of Binding Free Energies by MM/GBSA

The MM/GBSA method had been performed to calculate the binding free energies by using the single trajectory protocol. The 300 snapshots were extracted at a time interval of 10 ps from the last 3 ns of MD trajectories for the analysis of the binding free energy. The calculated binding free energies and components are listed in Table 1. Because the radius parameters of the fluorine, chlorine, bromine and iodine atoms are missing in the MM/GBSA module in Amber 12, we added radii of 1.39 Ǻ for fluorine, 1.75 Ǻ for chlorine, 1.85 Ǻ for bromine and 1.98 Ǻ for iodine to the pbsa program in Amber [17,18]. Table 1 lists the components of the molecular mechanics and solvation energies computed by MM/GBSA and the entropy contributions from the normal mode analysis. As seen in Table 1, the binding free energies of 1a, 1b, 1c and 1d to TPH1 are: −46.2, −38.0, −47.6 and −46.4 kcal·mol^−1^, respectively. Furthermore, it is encouraging that the ranking of the experimental binding free energies is consistent with our predictions, which shows that the current analyses by MM/GBSA method are reliable.

As shown in Table 1, both the intermolecular van der Waals and the electrostatics interactions are important contributions to the binding, whereas polar solvation terms oppose binding. Nonpolar solvation terms, which correspond to the burial of SASA upon binding, contribute slightly favorably. Although the gas-phase electrostatic values, $\Delta {\text{E}}_{\text{int}}^{\text{ele}}$ of the four complexes show that electrostatic interactions are in favor of the binding. However, the overall electrostatic interactions energies, $\Delta {\text{G}}_{\text{sol}}(\Delta {\text{E}}_{\text{int}}^{\text{ele}}+\Delta {\text{G}}_{\text{sol}}^{\text{ele}})$ are positive and unfavorable for the binding, which is caused by the large desolvation penalty of charged and polar groups that is not sufficiently compensated upon complex formation. Comparing the van der Waals/nonpolar ( $\Delta {\text{E}}_{\text{int}}^{vdw}+\Delta {\text{G}}_{sol}^{nopol}$) contributions with the electro-static contributions ΔG*_ele_*, we find that the association between inhibitor and TPH1 is mainly driven by van der Waals/nonpolar interaction in the complex than in solution. Although the electrostatic interactions between TPH1 and inhibitors are strong, the electrostatic interactions between the solvent (water molecules) and the ligand are much stronger. Thus, when a ligand transfers from the solvent to the binding pocket, the electrostatic contributions are unfavorable to ligand binding [19]. In addition, the contributions of the entropy changes to free energies (*T*Δ*S*) impair the bindings of inhibitors to TPH1. It is noted that $\Delta {\text{E}}_{\text{int}}^{vdw}$ values are highly correlated with the binding affinity ΔG*_bind_*; furthermore, $\Delta {\text{E}}_{\text{int}}^{vdw}$ is eight times more than $\Delta {\text{G}}_{sol}^{nopol}$. Therefore, van der Waals energies mostly drive the bindings of the inhibitors to TPH1. This result suggests that the optimizations of van der Waals interactions between the inhibitors and TPH1 may lead to the potent inhibitors.

### 2.3. Binding Mode of the TPH1–Inhibitor Complex

**Figure 4:**
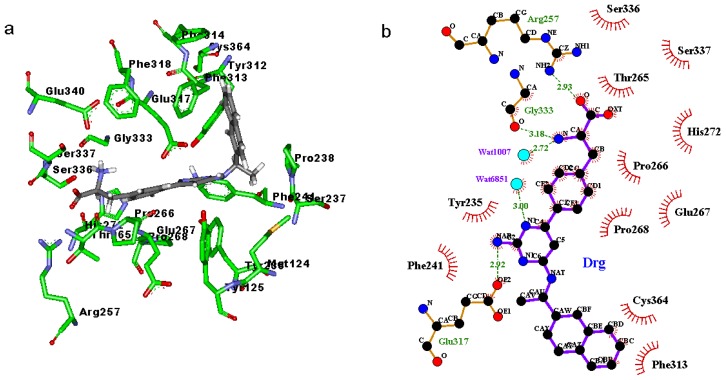
(**a**) Binding modes of inhibitor 1a with the key residues of TPH1 that are essential for the binding; (**b**) 2D contour of the binding modes generated by Ligplot plus.

**Figure 5:**
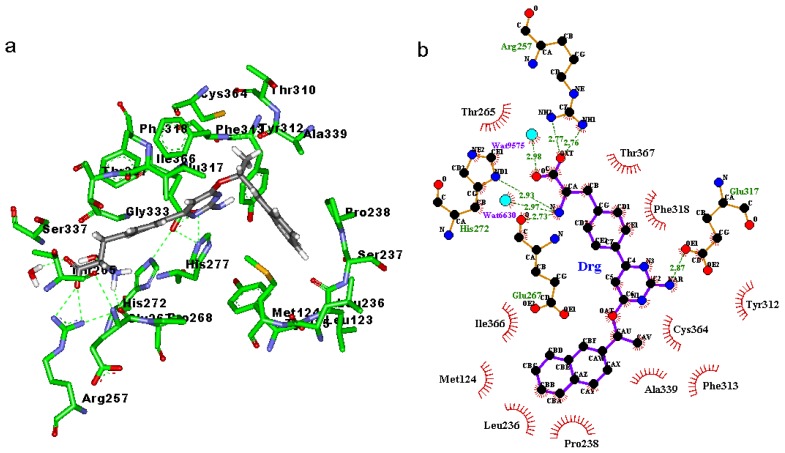
(**a**) Binding modes of inhibitor 1b with the key residues of TPH1 that are essential for the binding; (**b**) 2D contour of the binding modes generated by Ligplot plus.

**Figure 6:**
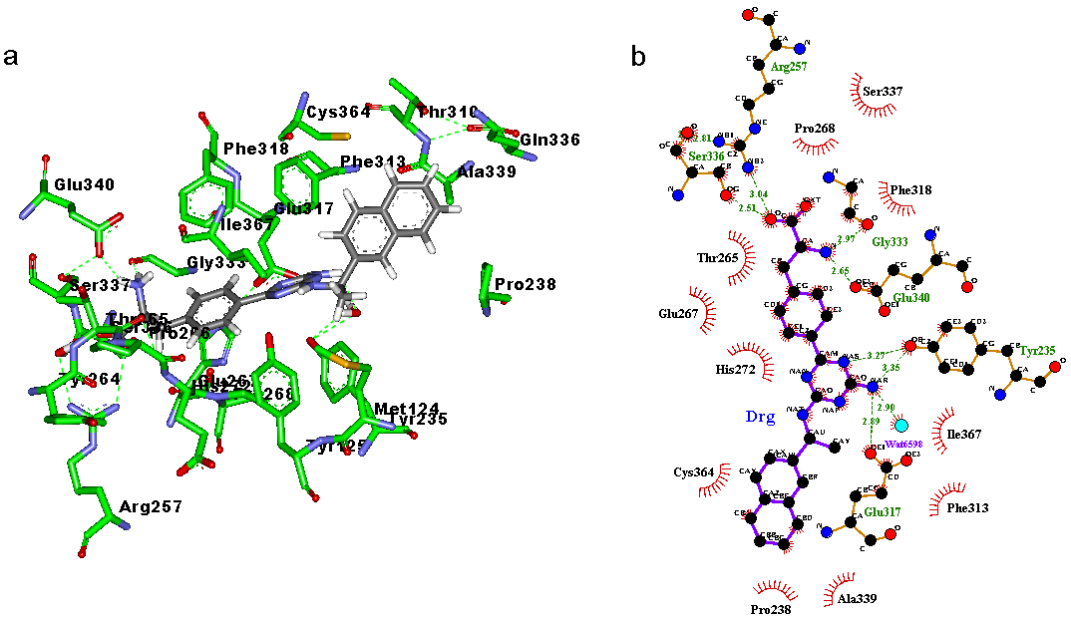
(**a**) Binding modes of inhibitor 1c with the key residues of TPH1 that are essential for the binding; (**b**) 2D contour of the binding modes generated by Ligplot plus.

**Figure 7:**
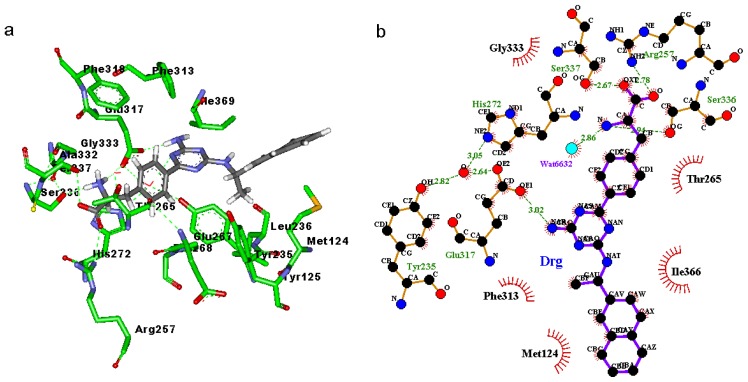
(**a**) Binding modes of inhibitor 1d with the key residues of TPH1 that are essential for the binding; (**b**) 2D contour of the binding modes generated by Ligplot plus.

Binding modes for the active site of TPH1 with inhibitors 1a to 1d are displayed in Figures 4 and 7. From Figures 4, 5, 6, 7a it can be observed that inhibitors extend deeply into the binding site of TPH1. The analysis of the intermolecular interactions including hydrophobic and hydrogen-bonding contacts on the TPH1–inhibitor complexes is carried out using Ligplot plus [20,21], as shown in Figures 4, 5, 6, 7b. The phenylalanine fragment could bind in a deep active site, formed by the hinge region residues (Arg257, Thr265, Glu267, His272, Glu333, Ser336 and Ser337) via two to five hydrogen bonds. The N atoms of the amino group could form hydrogen bonds with the backbone atoms of Gly333, Glu267 or side chain O atom of Ser336; the O and OXT atoms of the carboxyl group in the phenylalanine fragment could form hydrogen bonds with the side chain atoms of Arg257, Ser336 and Ser337, respectively. The 2-amino group of the pyrimidine or triazin ring could also form hydrogen bonds with the side chain atoms of Glu317 and Tyr235. Additionally, the oxygen atoms of the solvent water molecules could form other hydrogen bonds with the N atoms of inhibitors and backbone or side chains of the active site residues. These hydrogen bonds may help to stabilize the interaction between TPH1 and inhibitors. Moreover, the naphthalene ring could interact with a hydrophobic binding pocket, characterized by residues Met124, Leu236, Pro238, Phe313 and Ala339.

In order to further investigate the influence of the configuration on the hydrogen bonding network, the visible percentage of hydrogen bonds during the MD simulations was calculated and the results was displayed in Table 2. As shown in Table 2, according to Table 1, two significant results are obtained: (1) The hydrogen bonds between the carboxyl group and the active site residues, to inhibitors 1a, 1c and 1d, hydrogen bonds mainly existed between the O atom and the Arg257, but to inhibitor 1b, hydrogen bonds mainly existed between the OXT atom and the Arg257; (2) The hydrogen bonds between the 2-amino group of the pyrimidine or triazin ring and the active site residues, to inhibitors 1a, 1b and 1c hydrogen bonds mainly existed between the N atom and the Glu317, but to inhibitor 1d, hydrogen bonds mainly existed between the N atom and the Tyr235; and the occupied percentages of 1a, 1c and 1d were less than 20, but the occupied percent of 1b was more than 70. These two results showed that the inhibitors 1a and 1c can produce similar interaction contacts with TPH1. Moreover, the different hydrogen bond interaction modes between inhibitor 1b and the other inhibitors could come from its different binding conformation. Furthermore, it could be inferred that the different hydrogen bond interaction modes between inhibitor 1c and 1d may be from the chiral effect of the α-carbon atom of naphthalene ring.

### 2.4. Decomposition Analysis of the Binding Free Energies

**Figure 8:**
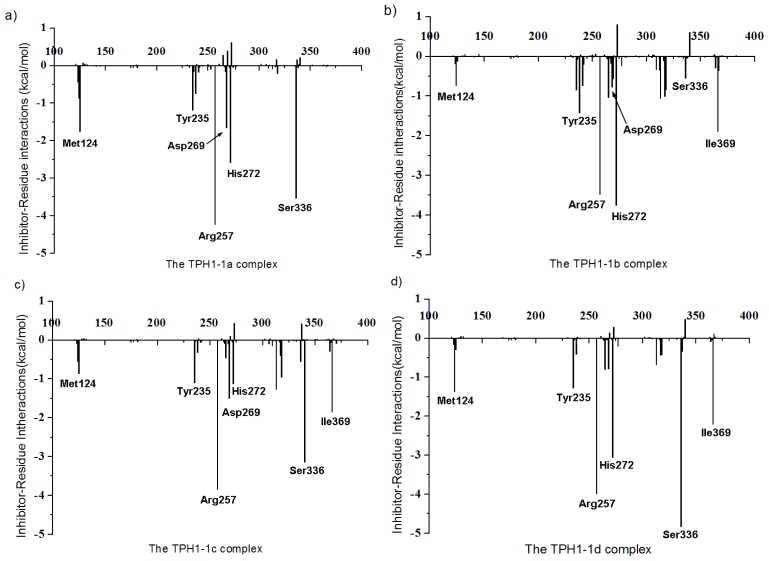
Decomposition of the binding free energies on a per-residue basis for the TPH1-1a (**a**), TPH1-1b (**b**), TPH1-1c (**c**) and TPH1-1d (**d**).

For the purpose of obtaining the detailed presentation of the inhibitor/TPH1 interactions, free energy decomposition analysis was employed to decompose the total binding free energies into inhibitor–residue pairs. The quantitative information of each residue’s contribution is extremely useful to interpret the binding modes of inhibitors with TPH1. The interactions between the inhibitors and each residue of TPH1 are plotted in Figure 8. In Figure 8, the four inhibitors have the similar interaction patterns, which mean stronger interactions with residue Met124, Tyr125, Tyr235, Pro238, Arg257, Glu267, Asp269, Thr370, Cys271, His272, Glu317, Gly333, Ser336 and Ile369 of TPH1. It is notable that Arg257, His272, Ser336 and Ile369 are the key residues for the distinction in all inhibitors.

### 2.5. Computational Mutagenesis of the Binding-Site Residues

**Figure 9:**
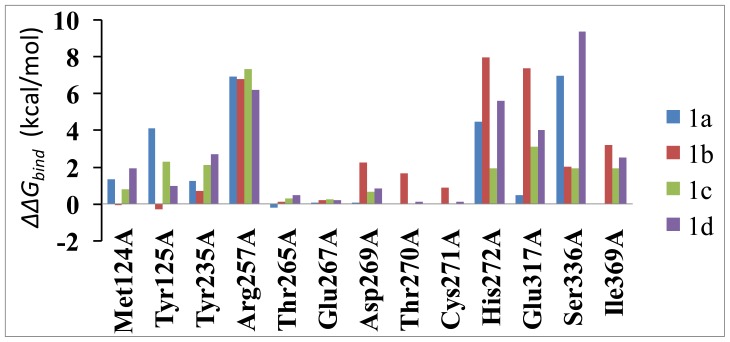
Effects of selected amino acid residues of the TPH1 binding pocket on the calculated free energies (kcal·mol^−1^) for the binding of four ligands to the TPH1 in the MM/GBSA computational alanine scanning (ΔΔ*G_bind_=* Δ*G_mut_* − Δ*G_wt_*).

To investigate other factors besides inter-molecular interactions that may help confer specificity, computational alanine scanning was employed to probe which residues make a significant intermolecular contribution to the differential in binding. This method depends on the assumption that local changes of the protein do not influence the whole conformation of the complex significantly. The thirteen key residues covering the walls of the pocket are mutated as shown in Figure 9 and the results of the mutagenesis are presented. Data are also depicted as a graph in Figure 9. It must be noted that computational mutagenesis was done with the single-trajectory method. This means that the simulation trajectory of the wild-type TPH1 complex was used to generate the structures of the mutated TPH1–inhibitor complexes. Positive energetic changes (ΔΔ*G_bind_=* Δ*G_mut_* − Δ*G_wt_*) represent an unfavorable interaction. As expected, we see that in general, mutations of active site residues are highly unfavorable with all four inhibitors. Figure 9 displays the changes of the inhibitor–residue interaction caused by the alanine scanning. The alanine scanning results in the reduction of the inhibitor–residue interaction energy for the selected residues. The inhibitor–residue interaction energies of six common residues have a decrease of higher 1.0 kcal·mol^−1^, and these residues include Tyr235, Arg257, His272, Glu317, Ser336 and Ile369. This result shows these six residues located in the hot spot of the surface between the inhibitor and TPH1. In addition, the decreases in the interaction energy of Tyr125 with inhibitor 1a is higher than 4.0 kcal mol^−1^, and the decreases in the interaction energy of Asp269, Thr270 and Cys271 with inhibitor 1b is higher than 1.0 kcal mol^−1^; the above results basically agree with the previous analyses.

## 3. Experimental Section

### 3.1. System Preparation

The four inhibitors of TPH1, which inhibitory activities were measured *in vitro* as the IC_50_ values, were obtained from previous work [7,8]. The chemical structures along with the experimental biological activities are shown in Figure 1. The crystal structure of TPH1 in complex with compound 1c (PDB entry: 3HF6, with the resolution of 1.8 Ǻ) was retrieved from the RCSB Brookhaven Protein Data Bank (PDB) [22]. The inhibitors 1a, 1b and 1d were constructed using the SYBYL-X 2.0 [23] molecular modeling software and were energy minimized with the Tripos force field. The missing hydrogen atoms of the inhibitors were added using SYBYL-X 2.0 while the missing atoms of 3HF6 were added using the *tleap* program in AMBER 12.0 [18]. The inhibitors were minimized using the Hartree–Fock (HF)/6-31G* optimi-zation in Gaussian09 [24], and the atom partial charges were obtained by fitting the electrostatic potentials derived by Gaussian via the RESP fitting technique in AMBER 12.0. The generations of the partial charges and the force field parameters for the inhibitors were accomplished by the antechamber program in AMBER 12.0. In the following molecular mechanics (MM) minimizations and MD simulations, the AMBER99 force field and the general AMBER force field (gaff) were used to establish the potential of proteins and inhibitors, respectively [25]. An appropriate number of chloride counter ions were placed around four TPH1–inhibitor complexes to neutralize the charges of the systems. Finally, the whole system was solvated in a cubic periodic box of TIP3P water molecules, and the distance between the edges of the water box and the closest atom of the solutes was at least 10 Å [26–28]. To avoid edge effects, periodic boundary conditions were applied during the whole molecular dynamics (MD) simulation.

### 3.2. Molecular Dymanics Simulation

For each system, energy minimization and MD simulation were performed by using the Sander module of the Amber 12. Prior to MD simulations, the entire system was subject to energy minimization in two stages to remove bad contacts between the complex and the solvent molecules. Firstly, the water molecules and counterions were minimized by freezing the solute using a harmonic constraint of a strength of 100 kcal·mol^−1^Å^−2^. Secondly, the entire system was minimized without restriction. Each stage consisted of a 5000-step steepest descent and a 2500-step conjugate gradient minimization.

In MD simulations, Particle Mesh Ewald (PME) was employed to deal with the long-range electrostatic interactions [29]. The cutoff distances for the long-range electrostatic and van der Waals energy interaction were set to 10 Å. The systems were gradually heated in the NVT ensemble from 0 to 300 K over 100 ps. Finally, 10 ns MD simulations were carried out for each system in an isothermal isobaric ensemble (NPT) with periodic boundary conditions. The SHAKE method [30], with a tolerance of 10^−5^ Å, was applied to constrain all covalent bonds involving hydrogen atoms. Each simulation was coupled to a 300 K thermal bath at 1.0 atm (1 atm = 101.3 kPa) by applying the Langevin algorithm [31]. The temperature and pressure coupling parameters were set as 1.0 ps. During the sampling process, the coordinates were saved every 0.1 ps and the conformations generated from the simulations were used for further binding free energy calculations and decomposition analysis.

### 3.3. MM/GBSA Calculation

Based on previous successful studies [32–34], we extracted a total number of 150 snapshots from the last 3 ns trajectory with an interval of 20 ps for binding free energy calculations. The MM/GBSA method and nmod module included in the Amber 12 were applied to compute the binding free energies of four inhibitors to TPH1 according to the following equation:

(1) $$\Delta G_{b}=\Delta {\text{E}}_{MM}+\Delta G_{\text{sol}}-\text{T}\Delta \text{S}$$

where (ΔE*_MM_* is the difference in molecular mechanics energy between the complex and each binding partner in the gas phase, Δ*G*_sol_ is the solvation free energy contribution to binding and TΔS is the contribution of entropy changes to the binding free energy. (ΔE*_MM_* is further divided into two parts:

(2) $$\Delta {\text{E}}_{MM}=\Delta {\text{E}}_{\text{int}}^{ele}+\Delta {\text{E}}_{\text{int}}^{vdw}$$

where $\Delta {\text{E}}_{\text{int}}^{ele}$ and $\Delta {\text{E}}_{\text{int}}^{vdw}$ are described as the electrostatic interaction and van der Waals energy in the gas phase, respectively. The solvation free energy is expressed as:

(3) $$\Delta G_{\text{sol}}=\Delta {\text{G}}_{sol}^{ele}+\Delta {\text{G}}_{sol}^{nopol}$$

The electrostatic contribution to the solvation free energy ( $\Delta {\text{G}}_{sol}^{ele}$) was calculated using the generalized Born (GB) model of Onufriev *et al.* [30]. The hydrophobic contribution to the solvation free energy ( $\Delta {\text{G}}_{sol}^{nopol}$) was determined with a function of the solvent-accessible surface area:

(4) $$\Delta {\text{G}}_{sol}^{nopol}=\gamma \text{SASA}+\beta$$

in which SASA is the solvent-accessible surface area and was calculated with the MSMS program [35]. In our calculations, the values for γ and β were set to 0.0072 kcal·mol^−2^ and 0 kcal·mol^−1^, respectively. The normal-mode analysis was performed to estimate the change of the conformational entropy upon the ligand binding (–TΔS) via the *nmode* program in AMBER 12.0. The protein–ligand binding free energy was calculated based on 300 snapshots taken from 7 to 10 ns MD simulation trajectories of the complex. Considering the high computational demand, only 50 snapshots for each complex were used to estimate the binding entropy.

### 3.4. Free Energy Decomposition Analysis

The interaction between inhibitors and each residue were computed using the MM/GBSA decomposition process by the *mm_pbsa* program in AMBER 12.0 [36]. The binding interaction of each inhibitor–residue pair includes three energy terms: van der Waals contribution (Δ*E*_vdw_), electrostatic contribution (Δ*E*_ele_), and solvation contribution (Δ*G*_GB_ + Δ*G*_SA_), in which Δ*E*_vdw_ and Δ*E*_ele_ are van der Waals and electrostatic interactions between the inhibitor and each protein residue that could be computed by the *Sander* program in AMBER 12.0 (University of California: San Francisco, CA, USA). The polar contribution of desolvation (Δ*G*_GB_) was calculated using the generalized Born (GB) model, whereby the parameters were developed by Onufriev *et al.* The nonpolar contribution of desolvation (Δ*G*_SA_) was computed based on SASA determined with the ICOSA method [18]. All energy components were calculated using 300 snapshots extracted from the MD trajectory from 7 to 10 ns.

### 3.5. Computational Alanine Scanning

To study the detailed mechanisms of the inhibitor–residue interaction at the energetic and atomic levels, computational alanine scanning was carried out on TPH1, and the binding free energies of the inhibitors to the protein mutants were calculated by using the MM/GBSA method. For alanine scanning, snapshots were generated every 10 ps from 7 to 10 ns in the wild type trajectory. Mutations to alanine were performed only on selected residues in the active site. Alanine mutations were generated by truncation of residues after the Cβ and adding a hydrogen atom in the same direction as the Cγ. Partial charges for the mutated residue were then changed to those of alanine. None of the residues mutated in this study were glycines. The binding free energy difference between the mutant and wild-type complexes is defined as

(5) $$\Delta \Delta G_{bind}=\Delta G_{mut}-\Delta G_{wt}$$

The polar contribution (Δ*G*_GB_) of $\Delta {\text{G}}_{sol}^{ele}$ was computed using the generalized Born model, and the parameters for GB calculations were developed by Onufriev *et al.* The charges used in GB calculations were taken from the AMBER parameter set. All energy components in Equation (5) were calculated using 300 snapshots taken from 7 to 10 ns of MD trajectory with the time interval of 10 ps. The key residues of TPH1: Met124, Tyr125, Tyr235, Arg257, Thr265, Glu267, Asp269, Thr370, Cys271, His272, Glu317, Ser336 and Ile369 were chosen for mutating. However, due to the significant difference in backbone conformations between proline and alanine, the Pro238 from the active site of TPH1 was not selected [37–39].

## 4. Conclusions

Ten nanoseconds of MD simulations and MM/GBSA calculations had been successfully run on four phenylalanine derivative inhibitor–TPH1 complexes. The analyses of dynamics properties involving RMSD, RMSF and structural superimposition show the stability of the complex during MD simulation. The calculation of binding free energy based on the MM/PBSA method was made, and the results prove that both van der Waals and electrostatics interactions drive the binding of four inhibitors to TPH1. We had performed the alanine scanning on four inhibitor–TPH1 complexes and computed their inhibitor–residue interactions. The results confirm that the hydrogen bond and hydrophobic interaction govern the inhibitor binding and six common residues located in the hot spot of the surface between the inhibitors and TPH1. Based on the free energy decomposition and structure analysis, the difference of the binding free energy is primarily determined by Tyr235, Arg257, His272, Glu317, Ser336 and Ile369. Additionally, it can be seen that the different configurations of the naphthalene group could form different binding patterns but result in similar binding affinity of compounds 1c and 1d. Thus, optimization of the hydrogen bond and van der Waals interactions between the hydrophobic groups of the inhibitors and the protein residues may lead to novel small molecule inhibitors that target the TPH1 protein. The results obtained from this study will be valuable for future rational design of novel and potent TPH1 inhibitors.

## Supplementary Information



## Figures and Tables

**Table 1:** Binding free energies and individual energy terms of inhibitors in complex with TPH1 (kcal/mol).

Contribution	1a	1b	1c	1d
$\Delta {\text{E}}_{\text{int}}^{ele}$	−49.8 (0.82)	−45.7 (0.82)	−47.3 (0.85)	−52.5 (0.55)
$\Delta {\text{E}}_{\text{int}}^{vdw}$	−42.8 (0.25)	−41.7 (0.45)	−49.3 (0.39)	−41.0 (0.37)
$\Delta {\text{G}}_{sol}^{nopol}$	−6.0 (0.02)	−7.677 (0.02)	−6.9 (0.03)	−6.6 (0.04)
$\Delta {\text{G}}_{sol}^{ele}$	66.9 (0.59)	68.837 (0.52)	69.2 (0.86)	66.7 (0.67)
ΔG*_sol_*^a^	60.9(0.58)	61.2 (0.51)	62.3 (0.84)	60.1 (0.59)
ΔG*_ele_*^b^	17.1 (0.38)	23.1 (0.53)	21.9 (0.58)	14.2 (0.43)
−TΔS	−12.6	−11.8	−13.3	−13.0
ΔG *_bind_*	−46.2 (0.41)	−38.0 (0.39)	−47.6 (0.56)	−46.4 (0.33)
IC_50_(nM)	32	380	26	44
Δ*G_exp_*^c^	−42.7	−36.6	−43.3	−42.0

a The polar/nonpolar ( $\Delta {\text{G}}_{sol}^{ele}+\Delta {\text{G}}_{sol}^{nopol}$) contributions;

b The electrostatic ( $\Delta {\text{E}}_{\text{int}}^{ele}+\Delta {\text{G}}_{sol}^{ele}$) contributions. All energies are averaged over 150 snapshots and are given in kcal/mol. Calculation of ΔG*_bind_* does not explicitly consider entropy contributions. The values in parentheses represent the standard error of the mean;

c Experimental binding free energies are calculated from IC_50_ using the following relationship: ΔG*_bind_* = RTlnK_dissociated_ = RTln (IC_50_ + 0.5C_enzyme_) ≈ RTlnIC_50_, where *R* is ideal gas constant, *T* is temperature in *K* (298 K is used in this article), and *C*_enzyme_ is the concentration of enzyme, which is a very small number after equilibration and can be omitted in most cases.

**Table 2:** Hydrogen bond analysis of the inhibitors into TPH1 binding site based on MD Simulation.

Inhibitor	Donor	AcceptorH	Acceptor	% Occupied	Distance (Ǻ)	Angle (Degree)
1a	DRG:O	257:HH22	257:NH2	98.3	2.832	21.92
	336:OG	DRG:H1	DRG:N	86.0	3.094	39.82
	333:O	DRG:H1	DRG:N	71.7	3.016	41.57
	317:OE2	DRG:HAR	DRG:NAR	17.3	3.046	18.62
	317:OE1	DRG:HAR	DRG:NAR	15.0	3.113	22.19
	DRG:O	265:HG1	265:OG1	11.7	3.003	31.25

1b	267:O	DRG:H1	DRG:N	80.7	2.899	30.02
	317:OE1	DRG:HAR	DRG:NAR	74.7	2.834	31.52
	DRG:OXT	257:HH22	257:NH2	66.0	2.847	19.37
	DRG:OXT	257:HH12	257:NH1	37.3	3.161	42.36
	DRG:O	257:HH22	257:NH2	27.0	2.823	22.22
	DRG:O	257:HH12	257:NH1	17.7	3.243	43.09
	267:O	DRG:H2	DRG:N	10.0	2.990	33.24

1c	DRG:O	257:HH22	257:NH2	87.0	2.844	18.14
	DRG:O	336:HG	336:OG	72.7	2.746	16.02
	333:O	DRG:H2	DRG:N	53.3	2.952	16.27
	340:OE2	DRG:H1	DRG:N	52.3	2.731	37.99
	340:OE2	DRG:H2	DRG:N	26.7	2.813	38.05
	333:O	DRG:H1	DRG:N	26.0	2.943	17.35
	DRG:O	257:HH12	257:NH1	20.0	3.224	43.44
	317:OE1	DRG:HAR	DRG:NAR	13.3	2.860	25.73
	DRG:OXT	257:HH12	257:NH1	10.0	3.227	24.00
	DRG:OXT	257:HH22	257:NH2	9.7	3.265	35.96
	235:OH	DRG:HAR	DRG:NAR	8.0	3.165	40.77

1d	DRG:O	257:HH22	257:NH2	99.3	2.800	18.60
	336:OG	DRG:H1	DRG:N	97.7	2.917	22.46
	DRG:OXT	337:HG	337:OG	32.3	2.655	19.75
	235:OH	DRG:HAR	DRG:NAR	17.3	3.192	41.50
	DRG:O	257:HH12	257:NH1	12.7	3.363	49.29
	333:O	DRG:H1	DRG:N	6.0	3.234	56.37

## References

[1] Gaspar P., Cases O., Maroteaux L. (2003). The developmental role of serotonin: News from mouse molecular genetics. Nat. Rev. Neurosci.

[2] Boyer E.W., Shannon M. (2005). The serotonin syndrome. N. Engl. J. Med.

[3] Martinez A., Knappskog P.M., Haavik J. (2001). A structural approach into human tryptophan hydroxylase and its implications for the regulation of serotonin biosynthesis. Curr. Med. Chem.

[4] Walther D.J., Peter J.U., Bashammakh S., Hortnagl H., Voits M., Fink H., Bader M. (2003). Synthesis of serotonin by a second tryptophan hydroxylase isoform. Science.

[5] Matthes S., Mosienko V., Bashammakh S., Alenina N., Bader M. (2010). Tryptophan hydroxylase as novel target for the treatment of depressive disorders. Pharmacology.

[6] Sikander A., Rana S.V., Prasad K.K. (2009). Role of serotonin in gastrointestinal motility and irritable bowel syndrome. Clin. Chim. Acta.

[7] Jin H.H., Cianchetta G., Devasagayaraj A., Gu K.J., Marinelli B., Samal L., Scott S., Stouch T., Tunoori A., Wang Y. (2009). Substituted 3-(4-(1,3,5-triazin-2-yl)-phenyl)-2-aminopropanoic acids as novel tryptophan hydroxylase inhibitors. Bioorg. Med. Chem. Lett.

[8] Shi Z.C., Devasagayaraj A., Gu K.J., Jin H., Marinelli B., Samala L., Scott S., Stouch T., Tunoori A., Wang Y. (2008). Modulation of peripheral serotonin levels by novel tryptophan hydroxylase inhibitors for the potential treatment of functional gastrointestinal disorders. J. Med. Chem.

[9] Ducy P., Karsenty G. (2010). The two faces of serotonin in bone biology. J. Cell Biol.

[10] Ducy P. (2011). 5-HT and bone biology. Curr. Opin. Pharmacol.

[11] Goltzman D. (2011). LRP5, serotonin, and bone: Complexity, contradictions, and conundrums. J. Bone Miner. Res.

[12] Yadav V.K., Balaji S., Suresh P.S., Liu X.S., Lu X., Li Z.S., Guo X.E., Mann J.J., Balapure A.K., Gershon M.D. (2010). Pharmacological inhibition of gut-derived serotonin synthesis is a potential bone anabolic treatment for osteoporosis. Nat. Med.

[13] Frost M., Andersen T., Gossiel F., Hansen S., Bollerslev J., van Hul W., Eastell R., Kassem M., Brixen K. (2011). Levels of serotonin, sclerostin, bone turnover markers as well as bone density and microarchitecture in patients with high-bone-mass phenotype due to a mutation in Lrp5. J. Bone Miner. Res.

[14] Inose H., Zhou B., Yadav V.K., Guo X.E., Karsenty G., Ducy P. (2011). Efficacy of serotonin inhibition in mouse models of bone loss. J. Bone Miner. Res.

[15] Camilleri M. (2011). LX-1031, a tryptophan 5-hydroxylase inhibitor, and its potential in chronic diarrhea associated with increased serotonin. Neurogastroenterol. Motil.

[16] Ouyang L., He G., Huang W., Song X., Wu F., Xiang M. (2012). Combined structure-based pharmacophore and 3D-QSAR studies on phenylalanine series compounds as TPH1 inhibitors. Int. J. Mol. Sci.

[17] Case D.A., Cheatham T.E., Darden T., Gohlke H., Luo R., Merz K.M., Onufriev A., Simmerling C., Wang B., Woods R.J. (2005). The Amber biomolecular simulation programs. J. Comput. Chem.

[18] Case D.A., Darden T.A., Cheatham T.E., Simmerling C.L., Wang J., Duke R.E., Luo R., Walker R.C., Zhang W., Merz K.M. (2012). AMBER 12.

[19] Yan C., Xiu Z., Li X., Li S., Hao C., Teng H. (2008). Comparative molecular dynamics simulations of histone deacetylase-like protein: Binding modes and free energy analysis to hydroxamic acid inhibitors. Proteins-Struct. Funct. Bioinforma.

[20] Laskowski R.A., Swindells M.B. (2011). LigPlot+: Multiple ligand-protein interaction diagrams for drug discovery. J. Chem. Inf. Model.

[21] Wallace A.C., Laskowski R.A., Thornton J.M. (1995). LIGPLOT—A program to generate schematic diagrams of protein ligand interactions. Protein Eng.

[22] Berman H.M., Westbrook J., Feng Z., Gilliland G., Bhat T.N., Weissig H., Shindyalov I.N., Bourne P.E. (2000). The protein data bank. Nucleic Acids Res.

[23] (2012). Sybyl-X Molecular Modeling Software Packages, Version 2.0.

[24] Frisch M.J., Trucks G.W., Schlegel H.B., Scuseria G.E., Robb M.A., Cheeseman J.R., Montgomery J.A., Raghavachari K., Vreven T., Kudin K.N. (2009). Gaussian 09, Revision C.01.

[25] Cheng Y.H., Cui W., Chen Q.A., Tung C.H., Ji M.J., Zhang F.S. (2011). The molecular mechanism studies of chirality effect of PHA-739358 on Aurora kinase A by molecular dynamics simulation and free energy calculations. J. Comput. Aided Mol. Des.

[26] Liu J., Liu M., Yao Y., Wang J., Li Y., Li G., Wang Y. (2012). Identification of novel potential beta-*N*-Acetyl-d-hexosaminidase inhibitors by virtual screening, molecular dynamics simulation and MM-PBSA calculations. Int. J. Mol. Sci.

[27] Zhang Y., Shen H., Zhang M., Li G. (2013). Exploring the proton conductance and drug resistance of BM2 channel through molecular dynamics simulations and free energy calculations at different pH conditions. J. Phys. Chem. B.

[28] Chen J., Zhang D., Zhang Y., Li G. (2012). Computational studies of difference in binding modes of peptide and non-peptide inhibitors to MDM2/MDMX based on molecular dynamics simulations. Int. J. Mol. Sci.

[29] Coleman T.G., Mesick H.C., Darby R.L. (1977). Numerical integration. Ann. Biomed. Eng.

[30] Darden T., York D., Pedersen L. (1993). Particle mesh Ewald: An N log (N) method for Ewald sums in large systems. J. Chem. Phys.

[31] Campanera J.M., Pouplana R. (2010). MMPBSA decomposition of the binding energy throughout a molecular dynamics simulation of amyloid-beta (Aβ10–35) aggregation. Molecules.

[32] Zhang H., Zan J., Yu G., Jiang M., Liu P. (2012). A combination of 3D-QSAR, molecular docking and molecular dynamics simulation studies of benzimidazole-quinolinone derivatives as iNOS inhibitors. Int. J. Mol. Sci.

[33] Lu S.-J., Chong F.-C. (2012). Combining Molecular docking and molecular dynamics to predict the binding modes of flavonoid derivatives with the neuraminidase of the 2009 H1N1 influenza a virus. Int. J. Mol. Sci.

[34] Mongan J., Simmerling C., McCammon J.A., David A., Onufriev A. (2007). Generalized Born model with a simple, robust molecular volume correction. J. Chem. Theory. Comput.

[35] Connolly M.L. (1983). Analytical molecular surface calculation. J. Appl. Cryst.

[36] Gohlke H., Kiel C. (2003). Case DA Insights into protein-protein binding by binding free energy calculation and free energy decomposition for the Ras-Raf and Ras-Ral GDS complexes. J. Mol. Biol.

[37] Hao M., Ren H., Luo F., Zhang S., Qiu J., Ji M., Si H., Li G. (2012). A computational study on thiourea analogs as potent MK-2 inhibitors. Int. J. Mol. Sci.

[38] Tong J., Chen Y., Liu S., Che T., Xu X. (2012). A descriptor of amino acids SVWG and its applications in peptide QSAR. J. Chemom.

[39] Tong J., Liu S. (2008). Three-dimensional holographic vector of atomic interaction field applied in QSAR of anti-HIV HEPT analogues. Qsar Comb. Sci.

