# Supplementary material for: Molecular Dynamics Simulation of Tryptophan Hydroxylase-1: Binding Modes and Free Energy Analysis to Phenylalanine Derivative Inhibitors
Source: Int J Mol Sci. 2013 May 10;14(5):9947–62. doi: 10.3390/ijms14059947 (PMC3676822; doi:10.3390/ijms14059947)
Supplement: Supplementary file 1 [file ijms-14-09947-s001.pdf]

Supplementary Information

Figure S1. Superimposition between the MD-simulated structure and the crystal structure: average structure is from the last 3 ns of MD simulation of the TPH1–inhibitor complex. Inhibitor 1a is displayed in the CPK mode1, the crystal structure of TPH1 is shown in cyan, MD-simulated structure of TPH1 to inhibitor 1a, 1b, 1c and 1d are shown in light grey, blue, yellow and magenta, respectively.

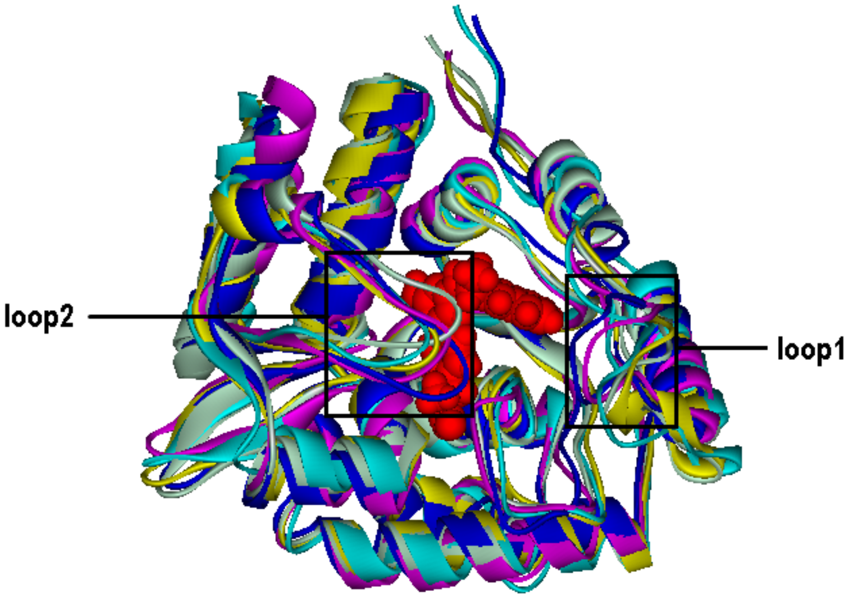

Figure S2. Superimposition of the MD-simulated structure of TPH1–inhibitor 1c and 1d complex.

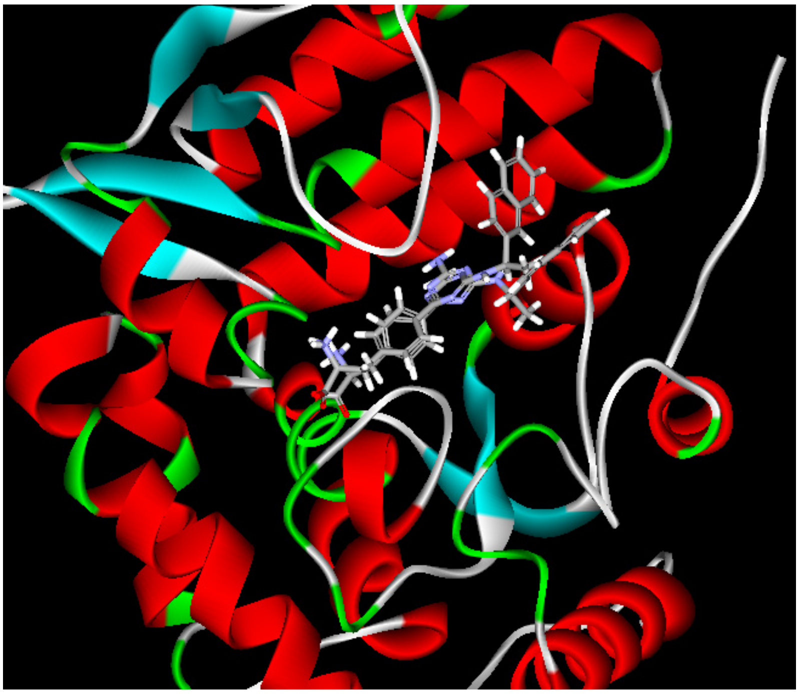

Figure S3. MD-simulated structure of TPH1–inhibitor 1c complex. The solvent accessible surfaces were generated by the binding site residues.

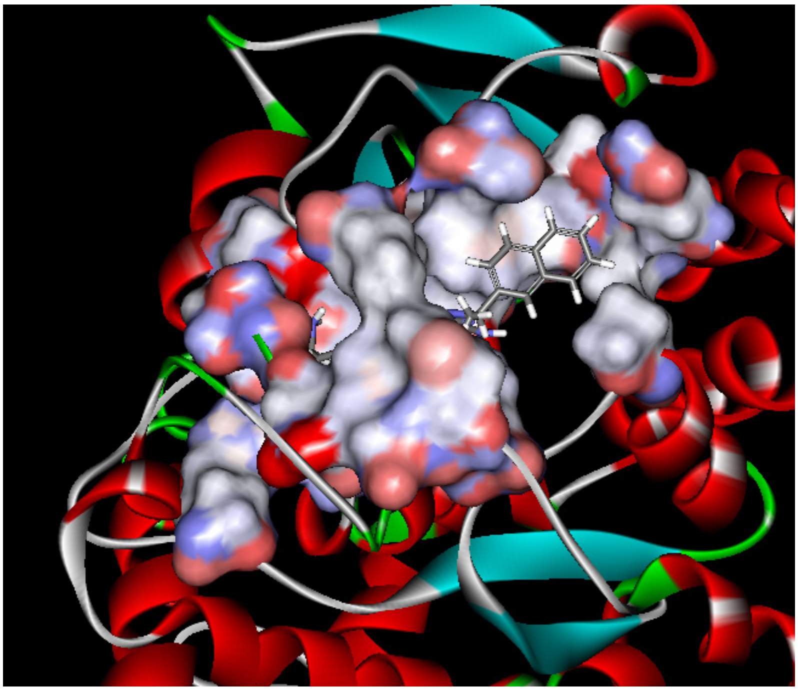

Figure S4. MD-simulated structure of TPH1–inhibitor 1d complex. The naphthalene ring of the inhibitor was stabilized by the hydrophobic interactions from Met124 and Ile369.

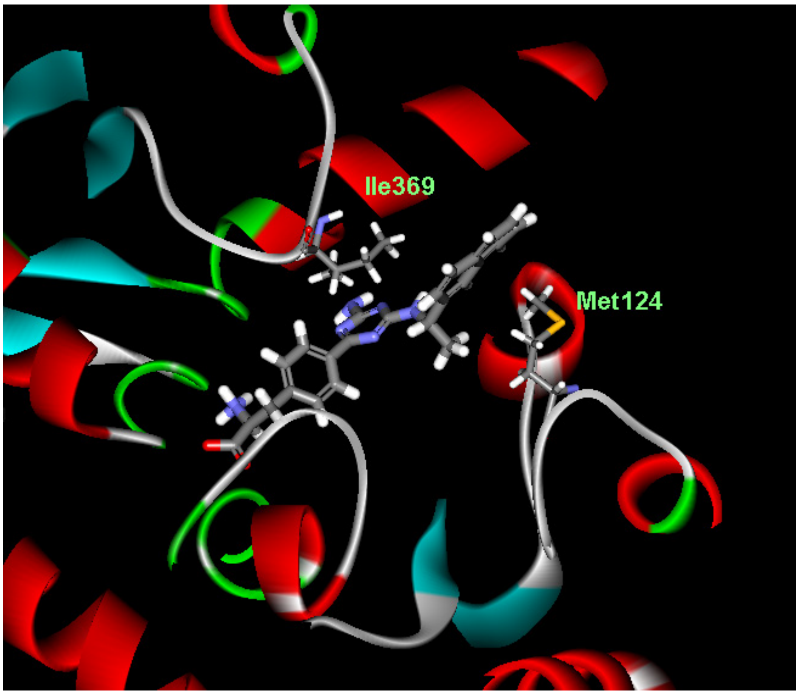

© 2013 by the authors; licensee MDPI, Basel, Switzerland. This article is an open access article distributed under the terms and conditions of the Creative Commons Attribution license (http://creativecommons.org/licenses/by/3.0/).
